# Updated Surveillance Metrics and History of the COVID-19 Pandemic (2020-2023) in Europe: Longitudinal Trend Analysis

**DOI:** 10.2196/53551

**Published:** 2024-06-21

**Authors:** Alexander L Lundberg, Scott A Wu, Alan G Soetikno, Claudia Hawkins, Robert L Murphy, Robert J Havey, Egon A Ozer, Charles B Moss, Sarah B Welch, Maryann Mason, Yingxuan Liu, Lori A Post

**Affiliations:** 1 Buehler Center for Health Policy and Economics Robert J. Havey, MD Institute for Global Health Northwestern University Chicago, IL United States; 2 Department of Emergency Medicine Feinberg School of Medicine Northwestern University Chicago, IL United States; 3 Feinberg School of Medicine Northwestern University Chicago, IL United States; 4 Department of Medicine, Division of Infectious Diseases Feinberg School of Medicine Northwestern University Chicago, IL United States; 5 Center for Global Communicable and Emerging Infectious Diseases Robert J. Havey, MD Institute for Global Health Northwestern University Chicago, IL United States; 6 Robert J. Havey, MD Institute for Global Health Feinberg School of Medicine Northwestern University Chicago, IL United States; 7 Department of Medicine, General Internal Medicine and Geriatrics Feinberg School of Medicine Northwestern University Chicago, IL United States; 8 Center for Pathogen Genomics and Microbial Evolution Robert J. Havey, MD Institute for Global Health Northwestern University Chicago, IL United States; 9 Institute of Food and Agricultural Sciences University of Florida Gainesville, FL United States

**Keywords:** Europe, COVID-19, history of the pandemic, method of the moments, Arellano-Bond estimators, Albania, Andorra, Austria, Belarus, Belgium, Bosnia and Herzegovina, Bulgaria, Croatia, the Czech Republic, Denmark, Estonia, Finland, France, Germany, Greece, Greenland, Hungary, Iceland, Ireland, the Isle of Man, Italy, Latvia, Liechtenstein, Lithuania, Luxembourg, Moldova, Monaco, Montenegro, the Netherlands, Norway, Poland, Portugal, Romania, San Marino, Serbia, Slovakia, Slovenia, Spain, Sweden, Switzerland, Ukraine, the United Kingdom, the Vatican City

## Abstract

**Background:**

In this study, we built upon our initial research published in 2020 by incorporating an additional 2 years of data for Europe. We assessed whether COVID-19 had shifted from the pandemic to endemic phase in the region when the World Health Organization (WHO) declared the end of the public health emergency of international concern on May 5, 2023.

**Objective:**

We first aimed to measure whether there was an expansion or contraction in the pandemic in Europe at the time of the WHO declaration. Second, we used dynamic and genomic surveillance methods to describe the history of the pandemic in the region and situate the window of the WHO declaration within the broader history. Third, we provided the historical context for the course of the pandemic in Europe in terms of policy and disease burden at the country and region levels.

**Methods:**

In addition to the updates of traditional surveillance data and dynamic panel estimates from the original study, this study used data on sequenced SARS-CoV-2 variants from the Global Initiative on Sharing All Influenza Data to identify the appearance and duration of variants of concern. We used Nextclade nomenclature to collect clade designations from sequences and Pangolin nomenclature for lineage designations of SARS-CoV-2. Finally, we conducted a 1-tailed *t* test for whether regional weekly speed was greater than an outbreak threshold of 10. We ran the test iteratively with 6 months of data across the sample period.

**Results:**

Speed for the region had remained below the outbreak threshold for 4 months by the time of the WHO declaration. Acceleration and jerk were also low and stable. While the 1-day and 7-day persistence coefficients remained statistically significant, the coefficients were moderate in magnitude (0.404 and 0.547, respectively; *P*<.001 for both). The shift parameters for the 2 weeks around the WHO declaration were small and insignificant, suggesting little change in the clustering effect of cases on future cases at the time. From December 2021 onward, Omicron was the predominant variant of concern in sequenced viral samples. The rolling *t* test of speed equal to 10 became insignificant for the first time in April 2023.

**Conclusions:**

While COVID-19 continues to circulate in Europe, the rate of transmission remained below the threshold of an outbreak for 4 months ahead of the WHO declaration. The region had previously been in a nearly continuous state of outbreak. The more recent trend suggested that COVID-19 was endemic in the region and no longer reached the threshold of the pandemic definition. However, several countries remained in a state of outbreak, and the conclusion that COVID-19 was no longer a pandemic in Europe at the time is unclear.

## Introduction

### Background

COVID-19, the disease caused by the virus SARS-CoV-2, was first detected in Wuhan, China, in the fall of 2019 [1-5]. The first European [6] case of COVID-19 was reported in France on January 24, 2020, with additional cases reported in Germany and Finland soon afterward [7,8]. Our research team conducted an analysis of the pandemic in Europe 1 year into the pandemic [9]; this study provides 2 additional years of updated surveillance and analysis for the region.

We adopt the World Bank’s definition of Europe, which is based on economic development and geographical proximity, encompassing Albania, Andorra, Austria, Belarus, Belgium, Bosnia and Herzegovina, Bulgaria, Croatia, the Czech Republic, Denmark, Estonia, Finland, France, Germany, Greece, Greenland, Hungary, Iceland, Ireland, the Isle of Man, Italy, Latvia, Liechtenstein, Lithuania, Luxembourg, Moldova, Monaco, Montenegro, the Netherlands, Norway, Poland, Portugal, Romania, San Marino, Serbia, Slovakia, Slovenia, Spain, Sweden, Switzerland, Ukraine, the United Kingdom, and the Vatican City [6].

The World Health Organization (WHO) and Director-General Tedros Ghebreyesus declared the end of COVID-19 as a public health emergency of international concern on May 5, 2023 [10-12], based on the recommendation of the COVID-19 Emergency Committee [12]. To that end, we compared how the pandemic was progressing before and after the declaration.

### Empirical Definition of Pandemic Versus Epidemic Versus Outbreak Versus Endemic

Epidemiological terms, such as pandemic, epidemic, outbreak, and endemic, are used to describe the occurrence and spread of diseases [13,14]. The distinctions between these terms lie in their scope, geographic extent, and severity. An epidemic refers to a sudden increase in the number of disease cases in a specific population or region. If the epidemic spreads across several countries or continents, it becomes a pandemic. An outbreak, on the other hand, describes a sudden increase in a concentrated setting, usually involving a more limited geographic area than an epidemic. Endemic refers to the constant presence of a disease in a particular geographic region or population, with no sudden increases in case volume [15,16].

### Traditional Surveillance Versus Enhanced Surveillance

Public health surveillance is the “ongoing, systematic collection, analysis, and interpretation of health-related data essential to planning and evaluation of public health practice” [17]. Surveillance not only explains the burden of death and disease due to a virus but also generates research questions and guides researchers on topics that require further investigation [18-32]. Surveillance allows us to compare the burden of disease between geographical regions and to understand which regions are most impacted. The impact can be measured through rates of how many people contract a disease and how many die, as well as the affiliated costs.

However, traditional surveillance carries several limitations that this study had addressed. Traditional surveillance provides a snapshot of what has already happened [18-32], meaning surveillance is static and only considers the past. In the middle of a burgeoning pandemic, policy makers and public health practitioners also need to understand what is about to happen. Is an outbreak increasing? Will growth switch from linear to exponential? Are more people dying from that particular condition in one place than another? To inform health policy and practice, knowledge of what is about to happen is often more valuable than knowledge of what did happen. To that end, we have developed enhanced surveillance metrics that reflect the dynamics of a pandemic and inform imminent growth, most importantly, where along the epidemiological outbreak curve a particular region is situated. We have also included dynamic metrics about the speed of the pandemic at the national, regional, and global levels and measured how the acceleration of speed this week compared to last week, as well as how novel infections last week would predict new cases this week. We can think of the latter measure as the echoing forward of cases. These metrics were tested and validated in prior research [9,33-43].

The novel metrics add acceleration, jerk, and 1-day and 7-day persistence to the traditional measure of speed. The rate of new COVID-19 cases per 100,000 population is the “speed” of the pandemic. Acceleration is the difference in speed from one unit of time to the next. A positive (negative) acceleration means cases are rising (falling), and an acceleration of 0 indicates an inflection point or stable speed. From physics nomenclature, “jerk” is the change in acceleration from one time interval to the next. A positive jerk may indicate explosive growth in a disease. Finally, 1-day and 7-day persistence measures capture the impact of the 1-day and 7-day lag of speed on current speed. These measures derive from an Arellano-Bond dynamic panel data model, and they capture the echo-forward effect of COVID-19 cases on future cases either 1 or 7 days later [44].

This research team used these metrics to effectively analyze the role of economic reopening on COVID-19 transmissions [42]. These metrics also provided the status of the pandemic in global regions, including Europe [9]. Finally, they helped quickly identify the emergence of the Omicron variant, and they were used in policy briefs throughout the pandemic [45].

For the purpose of this study, standard surveillance metrics explain what has already happened in Europe, while enhanced surveillance metrics speak to what is about to happen or where along an epidemiological curve a country may sit. We used both types of metrics to analyze the possible end to the pandemic.

### Objectives

This study has 3 objectives. First, we aimed to measure whether there was an expansion or contraction in the pandemic in Europe when WHO declared the end of the COVID-19 pandemic as a public health emergency of international concern on May 5, 2023. At both the region and country levels, we used advanced surveillance and analytical techniques to describe the status of the pandemic in a 2-week window around the WHO declaration. From a public health perspective, we need to know whether the rate of new COVID-19 cases was increasing, decreasing, or stable from week to week and whether any changes in the transmission rate indicated an acceleration or deceleration of the pandemic. Statistical insignificance is significant; it can signal the epidemiological “end” to the pandemic if the rate of new cases is 0 (or very low) and stable, meaning the number of new cases is neither accelerating nor decelerating.

Second, we used dynamic and genomic surveillance methods to describe the history of the pandemic in the region and situate the time window around the WHO declaration within the broader history. We included the ratio of COVID-19 deaths to the number of transmissions as a proxy for the mortality risk from infection at the population level. We also included a historical record of genomic surveillance from sequenced viral specimens to identify the appearance and spread of variants of concern in the region.

Third, we aimed to provide historical context for the course of the pandemic in Europe. We addressed several questions. How did countries respond to the pandemic? How did the region fare in terms of disease burden? Furthermore, what social, economic, and political factors shaped the course of COVID-19 in the region? This context can provide important lessons for disease prevention and mitigation in future pandemics.

## Methods

### Overview

This study conducted trend analyses with longitudinal COVID-19 data from Our World in Data [46]. This study provides updates of traditional surveillance data and dynamic panel estimates from the original study by Post et al [9,41,42,47,48]. For the region of Europe, the data comprised an unbalanced panel of 44 countries and territories, running from August 14, 2020, to May 12, 2023. Because a number of countries around the world switched from daily to weekly reports at various points in 2023, we used a cubic spline to interpolate daily new cases and deaths if any country had 4 consecutive periods of nonzero new cases interspersed by 6 days of 0 new cases.

To identify the appearance and duration of variants of concern, we also used data on sequenced SARS-CoV-2 variants from the Global Initiative on Sharing All Influenza Data (GISAID), which is an effective and trusted web-based resource for sharing genetic, clinical, and epidemiological COVID-19 data [49-52]. We used Nextclade nomenclature [53] to collect clade designations from sequences and Pangolin nomenclature for lineage designations of SARS-CoV-2 [54,55]. Metadata for the study period, which add geographic location to the clade designations, were collected on June 22, 2023. To avoid low-frequency or potentially erroneous samples, the data set was further filtered to exclude months with <100 available samples, variant groups with <5 samples in a month, and variant groups representing <0.5% of the total samples in a month. The final data set consisted of 184,386 total samples available on GISAID [49-52].

We analyzed the potential “statistical end” to the pandemic with a 1-tailed *t* test for whether the mean of speed was equal to or greater than the outbreak threshold of 10. We ran the test on a rolling 6-month window over weekly speed for the region, and we plotted the *P* values from the test over time. All statistical analyses were conducted in R (version 4.2.1; R Foundation for Statistical Computing) with the *plm* package (version 2.6-2) [47,56].

### Ethical Considerations

This study does not constitute research with human participants (as defined by 45CFR46:102) because all data are publicly available and contain no identifiable private information. The institutional review board’s review was therefore unsolicited.

## Results

### Dynamic Panel Estimates

Table 1 presents the dynamic panel estimates for the week of May 5, 2023.

**Table 1:** Arellano-Bond dynamic panel data estimates for COVID-19 dynamics at the country level in Europe for the week of May 5, 2023^a^.

Variable	Value	*P* value
1-day lag coefficient	0.404	<.001
7-day lag coefficient	0.547	<.001
Shift parameter week of April 28	0.032	.42
Shift parameter week of May 5	0.075	.48
Weekend effect	−0.272	.01

^a^Wald: *χ*^2^_6_=6104; *P*<.001; Sargan: *χ*^2^_540_=40; *P*>.99.

While the 1-day and 7-day lag coefficients were positive and statistically significant (*P*<.001 for both), they were moderate in magnitude (0.404 and 0.547, respectively). For example, the 7-day coefficient suggests a cluster effect in which 1 case on a given day predicts 0.547 cases 1 week later. The shift parameters for the weeks of April 28, 2023, and May 5, 2023, were small and statistically insignificant (*P*=.42 and *P*=.48, respectively), however, suggesting the cluster effect of cases remained stable around the window of the WHO declaration.

The dynamic panel estimates have several advantages over the basic reproductive number, R_0_, which estimates the average number of people a contagious person will infect [57]. Foremost, R_0_ depends on many variables, such as social distancing, vaccination rates, demographics, and the transmissibility of a pathogen. Because the SARS-CoV-2 virus has mutated over time, so has its R_0_, but rapidly updated estimates for R_0_ are difficult to obtain. Vaccination campaigns and public health mitigation efforts have also evolved and thereby shaped R_0_. The dynamic panel estimates are based on a recent, 120-day window, so they can quickly adjust to new circumstances. The Arellano-Bond model is also robust to time-invariant, unobservable factors (in the application, stable differences between countries); corrects for autocorrelation; and allows for statistical tests of various model parameters [42].

The Wald and Sargan tests can assess the validity of the dynamic panel model. The Wald hypothesis test checks whether the independent variables have explanatory power for the dependent variable. From Table 1, the Wald test was highly significant (*P*<.001), rejecting the null hypothesis of no explanatory power. The Sargan test checks the validity of the overidentifying restrictions of the model. A rejection of the null would be evidence against the validity, but the test failed to reject the null with *P*>.99.

### Statis Surveillance Metrics

Static surveillance metrics for the weeks of April 28 and May 5, 2023, are provided in Tables 2 and 3.

**Table 2:** Static COVID-19 surveillance metrics for European countries for the week of April 28, 2023.

Country	New COVID-19 cases, n	Cumulative COVID-19 cases, n	7-day moving average of new cases	Weekly transmission rate per 100,000 population	New weekly deaths	Cumulative deaths	7-day moving average of deaths	Death rate per 100,000 population	Conditional death rate
Albania	0	334,090	0	0	0	3604	0	0	0.01
Andorra	0	48,015	4.57	0	0	159	0	0	0
Austria	952	6,067,780	614.43	10.65	3	22,456	2.14	0.03	0
Belgium	317	4,795,866	256.43	2.72	5	34,280	4.57	0.04	0.01
Bosnia and Herzegovina	14	402,906	5.86	0.43	1	16,338	0.14	0.03	0.04
Bulgaria	155	1,305,030	132.57	2.29	3	38,339	1.86	0.04	0.03
Croatia	62	1,272,886	69.86	10.77	0	18,180	3.57	0.79	0.01
Czech Republic	93	4,640,818	86.43	0.89	0	42,778	1.71	0	0.01
Denmark	20	3,412,109	72	0.34	7	8572	5	0.12	0
Estonia	23	618,297	32.14	1.73	0	3001	0	0	0
Finland	279	1,475,841	246	5.04	8	9612	10.57	0.14	0.01
France	6161	38,946,430	4,651.43	9.09	57	163,076	35	0.08	0
Germany	1496	38,409,945	1,058.29	1.79	9	174,086	16.86	0.01	0
Greece	1763	6,031,868	1,870.86	118.81	0	36,811	8.14	0.53	0.01
Hungary	56	2,201,824	64.43	3.92	0	48,778	1.14	0.06	0.02
Iceland	0	209,191	0	0	0	260	0	0	0
Ireland	183	1,710,808	60.29	3.64	3	8914	2.43	0.06	0.01
Italy	3580	25,809,208	2,974.43	6.06	31	189,904	23.71	0.05	0.01
Latvia	11	977,734	17.43	0.59	0	6351	0.86	0	0.01
Liechtenstein	0	21,465	0	0	0	87	0	0	0
Lithuania	80	1,318,871	70.14	2.91	1	9671	1	0.04	0.01
Luxembourg	0	319,959	0	0	0	1232	0	0	0
Malta	9	118,524	12	11.94	0	835	0	0.19	0.01
Moldova	17	620,333	21	3.73	0	12,113	0.14	0.04	0.02
Monaco	2	16,763	1.71	5.48	0	67	0	0	0
Montenegro	24	291,702	26.71	3.83	0	2826	0	0	0.01
Netherlands	0	8,610,372	0	0	0	22,992	0	0	0
Norway	77	1,483,939	54.29	1.42	0	5476	4.57	0	0
Poland	251	6,514,536	136.29	0.63	2	119,559	1.14	0.01	0.02
Portugal	238	5,582,561	193.57	2.32	9	26,628	5.29	0.09	0
Romania	504	3,393,902	522.29	17.93	0	68,089	6	0.16	0.02
San Marino	15	24,189	10.71	44.52	0	125	0	0	0.01
Serbia	472	2,536,159	355.71	6.87	0	18,022	0.57	0	0.01
Slovakia	27	1,866,470	14.43	0	0	21,167	0	0	0.01
Slovenia	40	1,343,646	31.43	1.89	2	9336	3.14	0.09	0.01
Spain	1692	13,845,803	1,461.14	24.91	184	120,917	26.29	0.39	0.01
Sweden	140	2,707,171	147.71	9.31	0	24,174	10.71	0.71	0.01
Switzerland	134	4,403,491	142.29	10.75	0	14,004	0.71	0.08	0
Ukraine	1150	5,538,357	1,258.57	20.27	0	112,210	11.71	0.18	0.02
United Kingdom	1670	24,594,965	1,615.57	2.47	0	226,278	14.43	0	0.01

**Table 3:** Static COVID-19 surveillance metrics for European countries for the week of May 5, 2023.

Country	New COVID-19 cases, n	Cumulative COVID-19 cases, n	7-day moving average of new cases	Weekly transmission rate per 100,000 population	New weekly deaths	Cumulative deaths	7-day moving average of deaths	Death rate per 100,000 population	Conditional death rate
Albania	0	334,090	0	0	0	3604	0	0	0.01
Andorra	0	48,015	0	0	0	159	0	0	0
Austria	436	6,071,124	477.71	4.88	2	22,479	3.29	0.02	0
Belgium	184	4,797,400	219.14	1.58	1	34,300	2.86	0.01	0.01
Bosnia and Herzegovina	0	402,924	2.57	0	0	16,340	0.29	0	0.04
Bulgaria	86	1,305,755	103.57	1.27	1	38,351	1.71	0.01	0.03
Croatia	43	1,273,256	50.43	7.39	0	18,213	4.71	0.61	0.01
Czech Republic	74	4,641,292	67.71	0.71	2	42,788	1.43	0.02	0.01
Denmark	109	3,412,750	91.57	1.85	5	8619	6.71	0.09	0
Estonia	21	618,492	27.86	1.58	0	3001	0	0	0
Finland	205	1,477,339	214	3.70	6	9659	6.71	0.11	0.01
France	4657	38,973,507	3,868.14	6.87	28	163,242	23.71	0.04	0
Germany	1056	38,417,047	1,014.57	1.27	13	174,169	11.86	0.02	0
Greece	2048	6,044,517	1,878.29	138.05	0	36,863	7.43	0.50	0.01
Hungary	47	2,202,179	50.14	3.29	0	48,782	0.57	0.02	0.02
Iceland	0	209,191	0	0	0	260	0	0	0
Ireland	54	1,711,287	68.43	1.08	0	8917	0.43	0	0.01
Italy	2592	25,828,252	2,720.57	4.39	24	190,080	25.14	0.04	0.01
Latvia	9	977,805	10.14	0.49	1	6358	1	0.05	0.01
Liechtenstein	1	21,468	0.43	0	0	87	0	0	0
Lithuania	73	1,319,395	74.86	2.65	0	9672	0.14	0	0.01
Luxembourg	0	319,959	0	0	0	1232	0	0	0
Malta	7	118,574	7.29	9.71	0	835	0	0.19	0.01
Moldova	12	620,443	14.29	2.58	0	12,115	0.29	0.11	0.02
Monaco	2	16,773	1.43	5.48	0	67	0	0	0
Montenegro	5	291,817	16.43	0.80	0	2827	0.14	0	0.01
The Netherlands	0	8,610,372	0	0	0	22,992	0	0	0
Norway	59	1,484,287	49.71	1.09	0	5497	3	0	0
Poland	113	6,515,449	130.43	0.28	6	119,581	3.14	0.02	0.02
Portugal	231	5,583,979	202.57	2.25	3	26,671	6.14	0.03	0
Romania	408	3,397,365	461.43	14.52	0	68,117	4	0.13	0.02
San Marino	5	24,241	7.43	14.84	0	125	0	0	0.01
Serbia	272	2,538,142	283.29	3.96	1	18,033	1.57	0.01	0.01
Slovakia	21	1,866,647	24.57	0	0	21,167	0	0	0.01
Slovenia	27	1,343,831	26.43	1.27	1	9356	2.86	0.05	0.01
Spain	2502	13,845,803	2,143.29	36.82	0	120,917	0	0.25	0.01
Sweden	133	2,708,118	134.86	8.83	0	24,247	10.43	0.64	0.01
Switzerland	99	4,404,340	115.43	7.94	0	14,012	1.14	0.06	0
Ukraine	814	5,544,969	950.29	14.35	0	112,271	8.71	0.13	0.02
United Kingdom	1410	24,604,529	1,366.29	2.09	0	226,278	0	0	0.01

Most countries had a transmission rate considered low by the US Centers for Disease Control and Prevention [9,33-43,58]. Specifically, a “low” transmission is considered to be no more than 10 cases per 100,000 people per week. “Moderate” transmission is 10 to 50 cases per 100,000 people per week. “Substantial” transmission is 50 to 100 cases [58,59]. However, a number of countries were in a state of outbreak. In particular, Greece had a speed of 119 new cases per 100,000 population in the week of April 28, and San Marino had a speed of 45. Greece was therefore in a “substantial” outbreak, while San Marino was in a “moderate” outbreak. Austria, Croatia, Malta, Romania, Spain, Switzerland, and Ukraine were also in “moderate” outbreaks.

By the following week, only Greece, Romania, San Marino, Spain, and Ukraine remained in an outbreak. While speed dropped notably for San Marino, speed alarmingly increased for Greece and Spain. We noted that speed in island nations often vacillates between high and low rates of transmission.

Overall, the status of the pandemic around the WHO declaration in Europe is consistent with an “end” to the pandemic, but the distinction is muddied by continued outbreaks in several countries. The outbreaks in Greece and San Marino comprise a small portion of the overall European population, and speed is more variable in island nations. On the basis of the definition of a pandemic or an outbreak in several countries, the data suggest a shift from pandemic to endemic COVID-19, but the continued outbreaks in 3 other countries do caution that the conclusion may be premature.

Comparing Tables 2 and 3 demonstrates a drop in transmission rates before and after the WHO declared an end to COVID-19 as a public health emergency. Overall, the United Kingdom, Italy, and Germany had the most cases of COVID-19 transmissions and deaths, but these ranks are largely a function of population size. Thus, a better measure of COVID-19 fatality risk is the number of COVID-19 cases and deaths per 100,000 people. Moreover, death is often a better proxy for the state of an outbreak than transmissions because deaths are less likely to be undercounted [60]. Undercounting may be due to poor public health infrastructure, home antigen testing, or a dearth of polymerase chain reaction testing or other resources. When we control for the risk of death given the number of COVID-19 transmissions, we find that Eastern European countries, such as Bosnia and Herzegovina, Moldova, and Poland, had the highest conditional death rates. For example, Bosnia and Herzegovina had the highest conditional death rate of 0.04 deaths per confirmed case. These disparities could be driven by differences in public health infrastructure, demographics, and the influx of refugees from the Russian invasion of Ukraine.

### Enhanced Dynamic Surveillance Metrics

Tables 4 and 5 contain enhanced dynamic surveillance metrics for the weeks before and after May 5.

We note that the figures in Tables 4 and 5 are not calculated as day-over-day averages across the week, as they are in Tables 2 and 3. Thus, the magnitudes of speed differ slightly across the tables. Again, by the week of May 5, speed was low for every country except Greece, Romania, San Marino, Spain, and Ukraine. The 7-day persistence effect on speed was also relatively high for these countries but low for others. Acceleration was almost uniformly negative, with the exception of Greece and Spain, which saw their outbreaks grow somewhat from the first week to the second. Across the board, jerk tended to be very small in magnitude, suggesting little change in acceleration rates.

Table 6 compares the 7-day persistence effect on speed for the top 5 countries around the 2 weeks of the WHO declaration. These ranks largely reflect the speed in the countries with outbreaks in the prior tables.

**Table 4:** Novel surveillance metrics for European countries for the week of April 28, 2023.

Country	Speed^a^	Acceleration^b^	Jerk^c^	7-day persistence effect on speed^d^
Albania	0	0	0	0
Andorra	7.34	0	−2.15	2.81
Austria	6.87	0.42	1.22	5.08
Belgium	2.20	−0.12	−0.03	1.79
Bosnia and Herzegovina	0.18	0	0.06	0.19
Bulgaria	1.95	0.08	0.05	1.21
Croatia	12.12	−0.37	−0.05	7.44
Czech Republic	0.82	−0.07	−0.07	0.76
Denmark	1.22	−0.17	−0.17	0.84
Estonia	2.42	−0.02	0.11	1.70
Finland	4.44	−0.11	−0.15	2.73
France	6.86	−0.09	−0.35	4.92
Germany	1.27	0.02	0.01	0.88
Greece	126.11	−3.09	0.26	75.57
Hungary	4.52	−0.22	0.01	3.23
Iceland	0	0	0	0
Ireland	1.20	0.28	0.73	0.84
Italy	5.04	−0.48	−1.35	3.03
Latvia	0.94	−0.06	0	0.55
Liechtenstein	0.36	0	0	0.39
Lithuania	2.55	0	0.04	1.76
Luxembourg	0	0	0	0
Malta	15.67	−1.28	−0.01	12.24
Moldova	4.49	−0.30	0.03	4.01
Monaco	4.70	0.78	−0.78	0.85
Montenegro	4.26	0	0.21	2.97
Netherlands	0	0	0	0
Norway	1	0.03	0.04	0.67
Poland	0.34	−0.01	0.09	0.41
Portugal	1.88	−0.05	−0.32	0.99
Romania	18.59	−0.48	0.17	13.01
San Marino	31.80	2.97	5.94	23.16
Serbia	5.18	0.03	0.02	3.57
Slovakia	0.40	0	0	0.07
Slovenia	1.48	0.08	0.05	1.28
Spain	21.51	0.88	0.20	10.81
Sweden	9.80	−0.17	0	5.91
Switzerland	11.39	−0.17	−0.03	6.54
Ukraine	22.19	−0.56	−0.01	15.18
United Kingdom	2.39	−0.02	−0.05	1.32

^a^New COVID-19 cases per 100,000 people.

^b^The difference in speed from one week to the next.

^c^The change in acceleration from one week to the next.

^d^The impact of the 7-day lag of speed on current speed (the echo-forward effect of COVID-19 cases on future cases 7 days later).

**Table 5:** Novel surveillance metrics for European countries for the week of May 5, 2023.

Country	Speed^a^	Acceleration^b^	Jerk^c^	7-day persistence effect on speed^d^
Albania	0	0	0	0
Andorra	0	0	7.34	4.25
Austria	5.34	−0.82	−0.70	3.98
Belgium	1.88	−0.16	0.01	1.27
Bosnia and Herzegovina	0.08	−0.06	−0.06	0.10
Bulgaria	1.53	−0.15	−0.08	1.13
Croatia	8.75	−0.48	0.02	7.02
Czech Republic	0.65	−0.03	0.01	0.48
Denmark	1.56	0.22	0.17	0.71
Estonia	2.10	−0.02	0.09	1.40
Finland	3.86	−0.19	0.16	2.57
France	5.70	−0.32	0.11	3.97
Germany	1.22	−0.08	0.01	0.74
Greece	126.61	2.75	0.78	73.03
Hungary	3.50	−0.09	0.02	2.62
Iceland	0	0	0	0
Ireland	1.36	−0.37	−0.70	0.70
Italy	4.61	−0.24	0.29	2.92
Latvia	0.55	−0.02	0.12	0.55
Liechtenstein	1.09	0	0	0.21
Lithuania	2.72	−0.04	0.09	1.48
Luxembourg	0	0	0	0
Malta	9.82	−0.32	0.19	9.08
Moldova	3.04	−0.16	0.01	2.60
Monaco	3.91	0	0.78	2.72
Montenegro	2.62	−0.43	−0.43	2.47
Netherlands	0	0	0	0
Norway	0.91	−0.05	−0.01	0.58
Poland	0.33	−0.05	−0.08	0.20
Portugal	1.97	−0.01	0.09	1.09
Romania	16.43	−0.49	−0.10	10.77
San Marino	22.05	−4.24	−3.39	18.42
Serbia	4.12	−0.42	−0.05	3
Slovakia	0.45	0	0	0.23
Slovenia	1.25	−0.09	0.14	0.86
Spain	31.54	1.70	0.05	12.46
Sweden	8.95	−0.07	0.02	5.67
Switzerland	9.25	−0.40	−0.03	6.60
Ukraine	16.75	−0.84	0.02	12.85
United Kingdom	2.02	−0.06	0.03	1.39

^a^New COVID-19 cases per 100,000 people.

^b^The difference in speed from one week to the next.

^c^The change in acceleration from one week to the next.

^d^The impact of the 7-day lag of speed on current speed (the echo-forward effect of COVID-19 cases on future cases 7 days later).

**Table 6:** The European countries with the highest 7-day persistence estimate in the weeks of April 28 and May 5, 2023.

Rank	Country	7-day persistence week 1 (April 28)	7-day persistence week 2 (May 5)
1	Greece	75.57	73.03
2	San Marino	23.16	18.42
3	Ukraine	15.18	12.85
4	Romania	13.01	10.77
4	Spain	10.81	12.46
5	Malta	12.24	9.08

These metrics suggest that the pandemic may have ended in the region. Still, speed and persistence measures were moderate to high for several countries in outbreaks, and the region had not exited the pandemic with as much clarity as had several other global regions, such as South Asia or sub-Saharan Africa.

**Figure 1:**
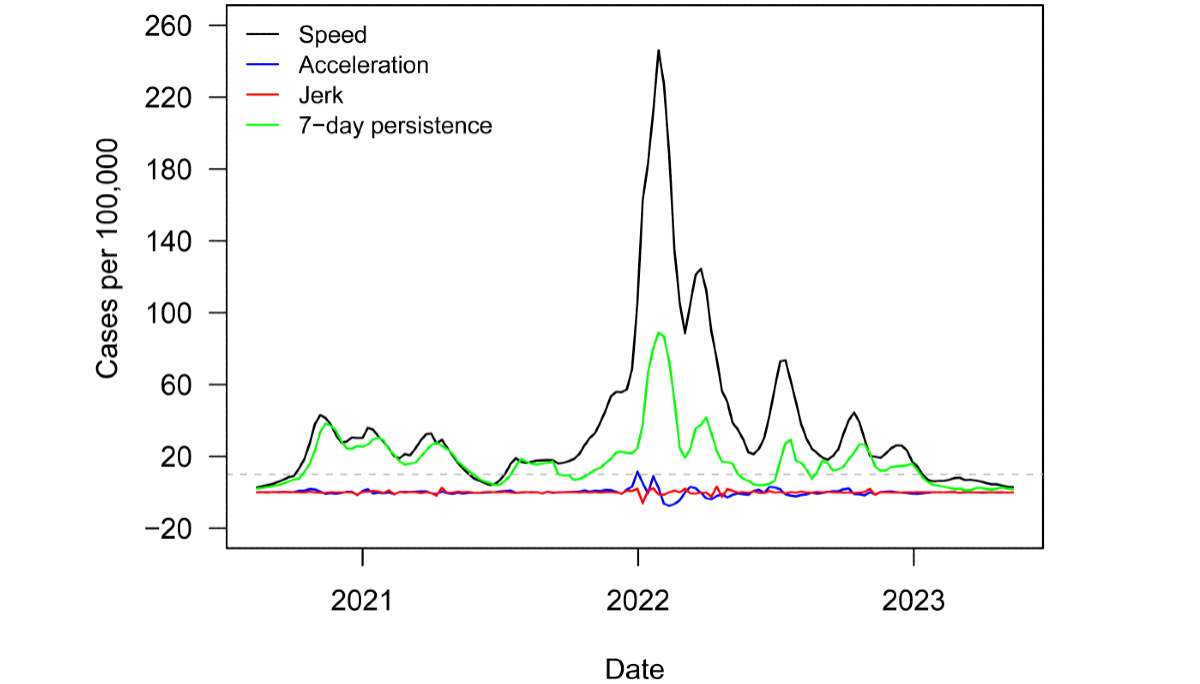
Novel surveillance metrics (speed, acceleration, jerk, and 7-day persistence) for COVID-19 transmissions in Europe from August 2020 to May 2023.

Figure 1 plots regional speed, acceleration, jerk, and 7-day persistence metrics from August 14, 2020, to May 12, 2023. The dashed gray line denotes the informal US Centers for Disease Control and Prevention outbreak threshold of speed equal to 10. The region was in a nearly continuous state of outbreak for the entire period. However, speed fell below the outbreak threshold and remained below it from January 2023 onward.

The region did see a slight bump in cases around the end of February 2023. Still, the bump did not cause the region to cross the outbreak threshold.

**Figure 2:**
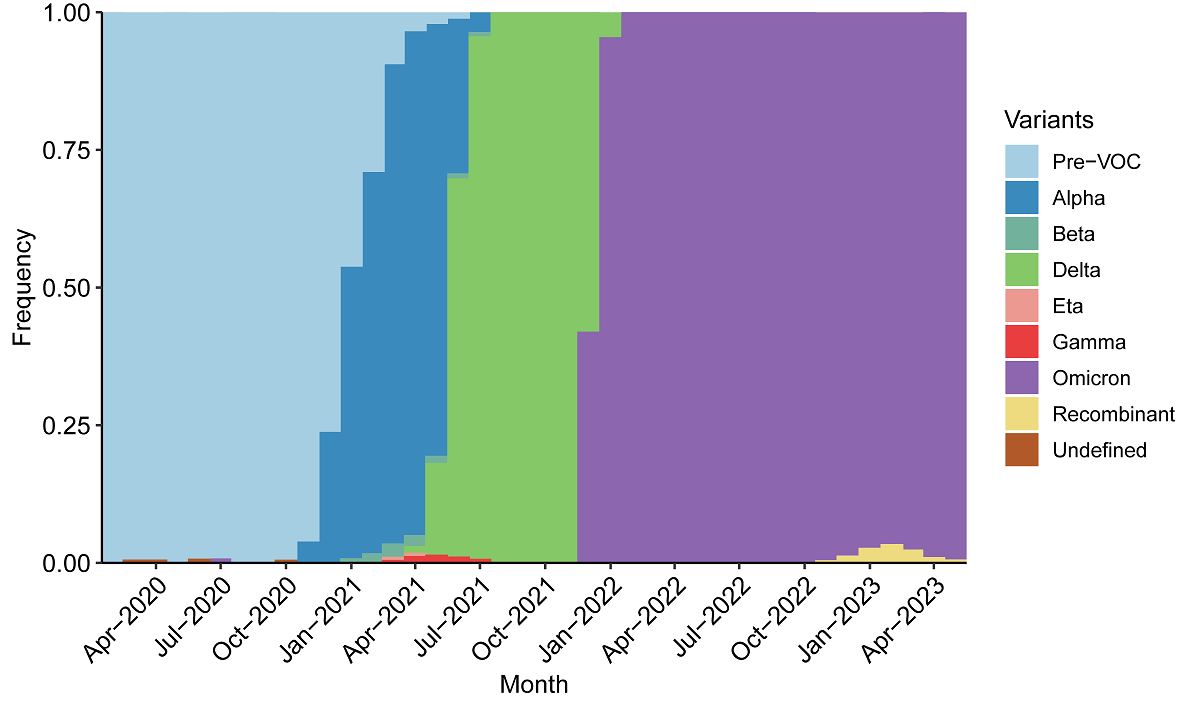
Variants of concern (VOCs) as a proportion of all sequenced SARS-CoV-2 specimens from April 2020 to May 2023 in Europe.

Europe saw one especially pronounced outbreak over the course of the pandemic. The outbreak caused speed to reach a peak of 246 novel COVID-19 cases per 100,000 population in the last week of January 2022. Figure 2 plots variant groups as a proportion of all viral specimens collected and sequenced in the region (and made available through GISAID) each month. The outbreak occurred just after the arrival of the Omicron variant. Europe, like much of the rest of the world, saw a surge in cases amid the heightened transmissibility of Omicron [45]. Earlier outbreaks were driven by the ancestral, Alpha, and Delta variants.

Another potential indication of the end of the pandemic is the continued dominance of the Omicron variant. Subclades of Omicron continue to circulate 4.5 months after WHO declared the end of the COVID-19 emergency [61]. Viral sequences have almost exclusively returned as Omicron and its subvariants ever since its arrival.

**Figure 3:**
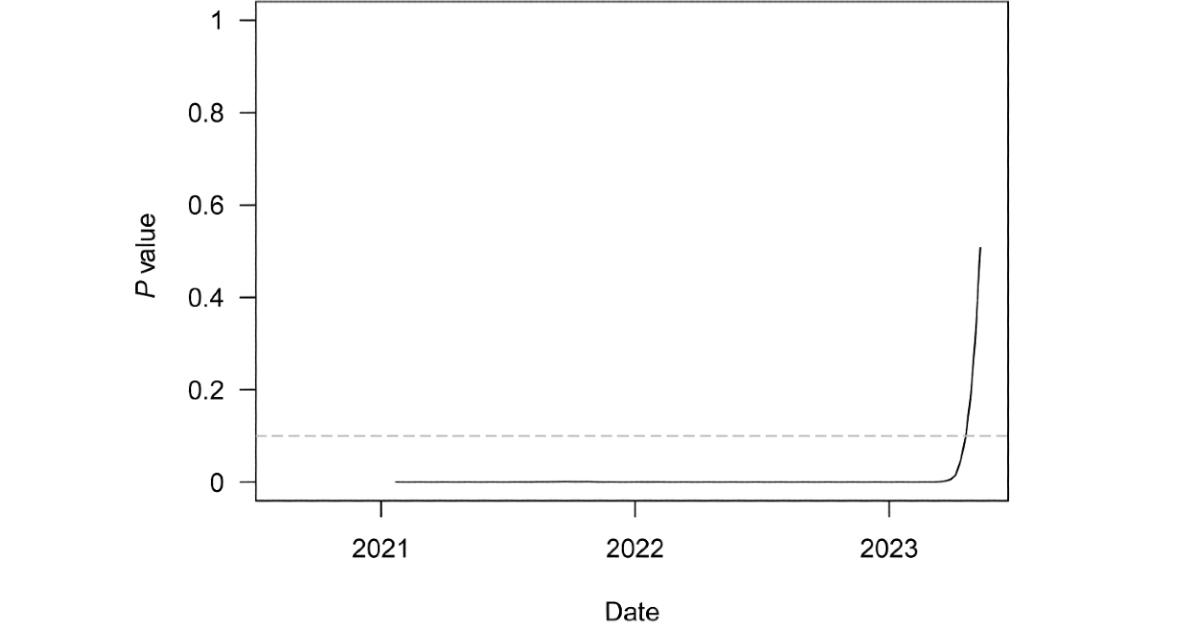
*P* values from t tests of weekly COVID-19 transmissions per 100,000 people equal to 10 over a rolling, 6-month window in Europe.

Figure 3 plots *P* values from a series of 1-tailed *t* tests of whether speed for the region was equal to or greater than the threshold outbreak of 10. These tests were conducted over a rolling 6-month window of weekly regional speed. The dashed gray line denotes the least restrictive conventional significance level threshold of α=.10. The test strongly rejected the null in favor of the alternative until the very end of April 2023. While this more recent lack of statistical significance is consistent with the end of the pandemic in the region, its relative recency around the WHO declaration suggests prematurity in the conclusion that COVID-19 had transitioned from the pandemic to endemic phase in Europe.

**Figure 4:**
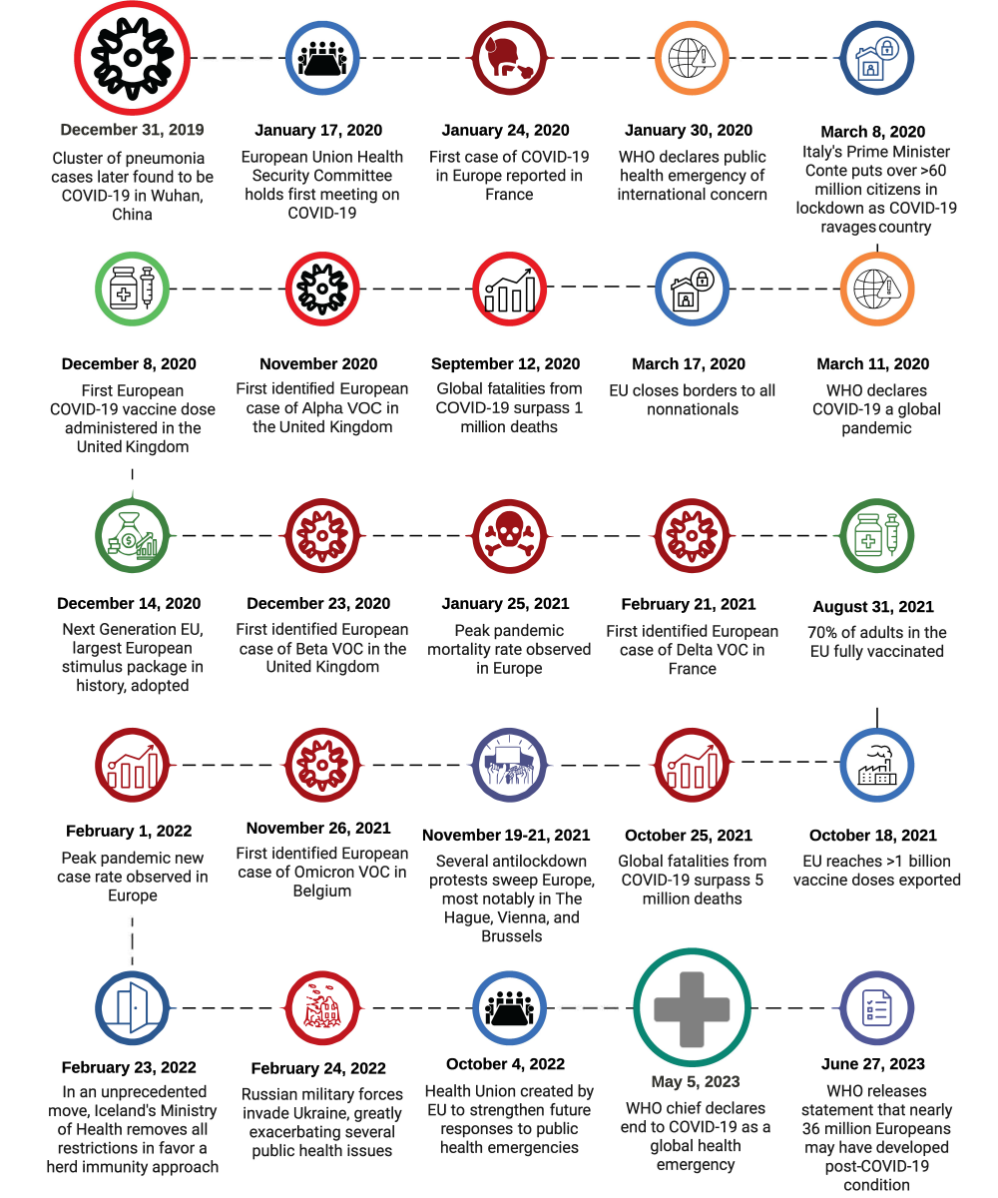
Timeline of the COVID-19 pandemic in Europe. EU: European Union; VOC: variants of concern; WHO: World Health Organization.

Figure 4 provides a time line of the onset of COVID-19 in Europe as well as vaccination programs and major events that likely shaped the course of disease control, such as the Next Generation European Union (NGEU) economic recovery package and the Russian invasion of Ukraine. Millions of refugees fled Ukraine, accelerating the spread of disease in the region. Mass human migration is affiliated with increased disease transmission [62].

## Discussion

### Principal Findings

Multiple metrics suggest the pandemic had largely subsided in Europe by the time of the WHO declaration. Regional speed had remained below outbreak status for 4 months, and acceleration and jerk were both low and stable. The 1-day and 7-day persistence coefficients did remain statistically significant (*P*<.001 for both), but the coefficients were moderate in magnitude, and the shift parameters for the weeks around the WHO declaration were insignificant. This statistical insignificance implies no change in the clustering effect of cases on future cases at the time. The rolling *t* test of speed equal to 10 became insignificant for the first time in the month ahead of the declaration. Finally, Omicron had been the predominant variant of concern in sequenced viral samples for approximately 6 months.

Still, among the 44 countries in Europe, 5 had surpassed the outbreak threshold at the time of the WHO declaration. While the region as a whole did not breach the outbreak threshold, 5 countries were classified as having epidemic status, with 4 being categorized as having mild epidemics. Our analysis of transmission rates, outbreak testing, and statistical findings suggests that the pandemic is likely concluding in Europe. Nevertheless, exercising caution, we refrain from definitively declaring the end of the pandemic at the time of the WHO declaration.

### History of Policy and Disease Burden

For a brief history of policy and disease burden, differences in health outcomes across countries remained relatively minor despite heterogeneity in European COVID-19 mitigation efforts in terms of school closures, business restrictions, quarantines, social distancing, and mask mandates [63]. For instance, Denmark implemented the most stringent measures, while Sweden pursued a comparatively relaxed approach [63,64]. The environment and climate factors also factored into differential transmission and death rates [64,65]. Notably, widespread vaccination efforts proved effective in reducing the severity of COVID-19 cases and lowering mortality rates [66].

Age structure and environmental risk factors such as pollution affected the COVID-19 pandemic at its onset [67]. Italy reported their first cases in March 2020 [68-70]. Hospitals and cities lacked preparedness, with the initial wave claiming 35,000 lives [68]. Similar challenges were observed in France, which struggled despite having robust health care resources [7,68,71-74]. Around 6 months into the pandemic, France had one of the highest prevalence rates of the virus in Europe, with >2 million cumulative confirmed cases at the time [73]. Despite benefiting from a universal health insurance system, centralized presidential regime with a strong public administration, and a relatively high number of health care professionals and hospital beds compared to other European countries, France was still underprepared for the pandemic’s onset, with the health care system soon overwhelmed by cases and fatalities [73,75]. During the peak months in 2020, Spain, Belgium, and Ireland had the highest incidence and mortality rates [76]. European health care systems improved as vaccines became available [77]. Despite difficulties, Europe’s health care system benefited from risk recognition, treatment availability, and increased vaccine access [78-81]. Throughout the pandemic, new variants and fluctuating social protection measures have led to additional waves of infection [82,83]. Since this time, case rates have fallen despite mild rebound periods, with the COVID-19 social and financial recovery in Europe benefiting from rapidly decreased social restrictions and unprecedented monetary investments [84].

### Policies Implemented to Control and Mitigate the Transmission of COVID-19

When the WHO declared COVID-19 a pandemic [85], Europe was the epicenter [86]. The European Union (EU) responded by closing external borders [7,87], but public reactions to social restrictions were volatile, leading to protests [88,89]. Despite this, Europe adapted to new regulations and accelerated its digital transformation [90,91].

The pandemic caused a 4% economic decline in Europe in 2020, with wide variation between countries [92]. To aid response, the EU allocated funds, including €140 million (US $159.6 million) in emergency funding [93]. Member states received continuous support through programs such as European instrument for temporary Support to mitigate Unemployment Risks in an Emergency [94,95]. In addition, in July 2020, the European Council, the European Commission’s sibling executive arm in the EU, agreed to an unprecedented €750 billion (US $855 billion) recovery package titled NGEU to be disseminated to member states through the end of 2023 [96]. NGEU, the largest stimulus package in European history, included special investments in future health care preparedness and modernization [97].

The EU’s Health Security Committee held its first meeting regarding COVID-19 on January 17, 2020, with the first mobilization of COVID-19 research funds taking place on January 31, 2020 [94]. Early efforts were made in conjunction with member states to secure medical equipment and protective gear for health care workers and ensure the free movement of critical workers to and around the EU [94]. Lockdowns and travel restrictions put in place by mid-March 2020, which is approximately when Italy’s total death toll overtook China during the first wave of the pandemic, affected >250 million Europeans [98]. As the pandemic intensified in the spring and summer of 2020, the European Commission partnered with Global Citizen and the European Investment Bank to develop tests, treatments, and vaccines with >€10 billion (US $11.4 billion) in new funds [94]. The most stringent virus mitigation orders in the United Kingdom came from Prime Minister Boris Johnson and the House of Commons in late March 2020, which granted the prime minister emergency powers to enforce widespread lockdowns and travel restrictions [99].

### Vaccination Campaigns

Europe’s vaccination strategy featured early collective bargaining, resulting in 2.8 billion vaccine doses secured for member states through an advance purchase agreement in June 2020 [93]. The United Kingdom administered its first vaccine dose on December 8, 2020 [100], and by August 2021, the EU had fully vaccinated 70% of its adult population, exporting >1 billion vaccine doses to low- and middle-income countries by October 2021 [94]. However, some Eastern European countries, such as Hungary, Poland, Romania, and Bulgaria, lagged behind with <65% of their populations fully vaccinated by February 2022 [101].

In total, 4 vaccines received emergency use authorization and were widely used in Europe throughout the pandemic: BioNTech or Pfizer, Moderna, Oxford or AstraZeneca, and Johnson & Johnson [102-104]. Vaccine development, approval, and rollout occurred in rapid fashion due to collective efforts by regulatory agencies, private pharmaceutical companies, public universities, and public health agencies [102].

### Humanitarian Crises

During the Omicron surge in early 2022, Russia’s attack on Ukraine displaced two-thirds of Ukrainians, leading to surges in Omicron and other infectious diseases, including tuberculosis. This conflict hindered COVID-19 vaccine distribution and access to essential medical services, such as HIV treatment [105-107]. Ukrainians faced obstacles in migrating without vaccination proof, as only 36% were vaccinated when the war began [108-113].

Europe absorbed many refugees during the COVID-19 pandemic, including those fleeing political unrest and violence in the Middle East and Eastern Europe [114,115]. In addition to overcrowded and unsafe living conditions and limited access to financial or medical support, many migrants were unable to access timely COVID-19 vaccination [114,115]. Some countries, like Spain, prioritized migrant vaccination, while Portugal granted temporary residence to ensure equal access [115].

### Limitations

The COVID-19 data had become less frequently reported around the world by the time the WHO declared an end to the pandemic public health emergency [116]. In addition, more people began to use at-home tests as the pandemic evolved [117], and the Russian invasion of Ukraine damaged public health infrastructure, which may have reduced the accuracy of reported cases in the region. Because the enhanced surveillance metrics of speed, acceleration, jerk, and 7-day persistence are based on rates, not total counts, statistical bias caused by countries dropping in or out of the sample is mitigated, but to the extent that a nonincluded country is unrepresentative of the region in disease burden; the omission of a country or territory can still influence historical data comparisons. Viral specimen tests for variants of concern in GISAID are also dependent on testing and sequencing capacity, which varied by country across the region.

### Conclusions

While there is significant evidence indicating that the pandemic in Europe has transitioned to an endemic phase, the persistent risk of new COVID-19 variants underscores the need for vigilance, robust vaccination campaigns, and international cooperation to effectively curb the spread of coronavirus in the region [40]. As the data on transmissions become less frequent [117] and as pandemic fatigue grows [118], the challenge of vigilance also evolves. The public health lessons from European policy and disease burden can inform not only the continued challenge but also responses to inevitable future pandemics.

## Abbreviations

EU: European Union
GISAID: Global Initiative on Sharing All Influenza Data
NGEU: Next Generation European Union
WHO: World Health Organization

## Data Availability

The data sets analyzed during this study are available in the Our World in Data repository [46] and the Global Initiative on Sharing All Influenza Data repository [49].
